# The Prognostic Nutritional Index before durvalumab after chemoradiation predict the overall survival in patients with stage III non-small cell lung cancer

**DOI:** 10.1080/07853890.2023.2196089

**Published:** 2023-04-12

**Authors:** Shun Matsuura, Sayaka Serizawa, Ryoma Yamashita, Keisuke Morikawa, Yutaro Ito, Toshiya Hiramatsu, Eisuke Mochizuki, Kazuki Tanaka, Norimichi Akiyama, Masaru Tsukui, Naoki Koshimizu, Takashi Kosugi

**Affiliations:** a Division of Respiratory Internal Medicine, Fujieda Municipal General Hospital, Fujieda, Japan; b Department of Radiation Oncology, Fujieda Municipal General Hospital, Fujieda, Japan

**Keywords:** Stage III non-small cell lung carcinoma, malnutrition, prognostic nutritional index

## Abstract

**Background:**

Adjuvant durvalumab after chemoradiation has become the standard of care for patients with stage III NSCLC, according to the PACIFIC trial. Whether biomarkers before durvalumab for patients with stage III NSCLC showed predictive and prognostic effects remains unknown.

**Methods:**

This is a retrospective study in the Fujieda Municipal General Hospital between October 2018 and March 2022. We assessed the predictive value of the Prognostic Nutritional Index (PNI) in stage III non-small cell lung cancer (NSCLC) patients treated with durvalumab after chemoradiation.

**Results:**

After applying the inclusion and exclusion criteria, the study included 56 patients for further analysis. The median follow-up period was 17.6 months (range, 3.0–45.4 months). According to receiver operating characteristic curve results, the PNI cutoff value to predict overall survival (OS) was 37.9, with sensitivity and specificity at 67.9% and 67.9%. Accordingly, the patients were divided into low- and high-PNI groups. Patients with the low-PNI group had a significantly shorter progression-free survival compared to the high-PNI group (median, 9.1 vs. 21.3 months, *p* = 0.032). OS was also shorter in the low-PNI group (median, 19.0 months vs. not reached, *p* < 0.001). In the multivariate Cox hazards regression analyses, the high-PNI was an independent prognostic factor for OS (hazard ratio, 0.187; 95% confidence interval, 0.046–0.760; *p* = 0.019).

**Conclusions:**

It seems that PNI could be used as a predictor for OS in patients with stage III NSCLC treated with durvalumab after chemoradiation.

**KEY MESSAGES**
- Inadequate immunocompetence and nutritional status after chemoradiation therapy may result in poor antitumor efficacy of ICIs.
- Pretreatment immune and nutritional assessment using PNI could be considered an independent predictor for the survival of stage III NSCLC patients treated with durvalumab after chemoradiation therapy.

## Introduction

Patients with stage III non-small cell lung cancer (NSCLC) are treated with platinum-based doublet chemotherapy and radiotherapy; however, chemoradiation alone has a poor prognosis [1]. Adjuvant durvalumab after chemoradiation has become the standard of care for patients with stage III NSCLC, according to the PACIFIC trial [2]. Predicting treatment efficacy with durvalumab for patients with stage III NSCLC is critical for clinical administration and maintenance decisions.

The progression of lung cancer is closely linked to cancer-related inflammation and nutrition. PNI, which is calculated by the count of peripheral lymphocyte and albumin concentration [3], is widely accepted as an index for nutritional and inflammatory conditions and it correlates with therapeutic efficacy and long-term outcome in various advanced malignancies [4–7]. It is unclear, whether PNI can be used to predict the therapeutic efficacy of immune check inhibitors (ICI) after chemoradiation therapy in stage III NSCLC. We hypothesized that PNI value after chemoradiation therapy and before durvalumab treatment could predict progression-free survival (PFS) and overall survival (OS) in stage III NSCLC patients.

## Patients and methods

### Study population

This retrospective study included 56 patients with stage III NSCLC who received durvalumab after chemoradiation therapy. The patients were selected from the Fujieda Municipal General Hospital between October 2018 and March 2022, and the ethical committee of the same hospital approved the study conduction (R04-22). The patients were included in the study only if they were (a) ≥18 years old, (b) clinically diagnosed as stage IIIA, IIIB, or IIIC according to the eighth edition of the International Union Against Cancer Tumor, Node, Metastasis Classification was used to stage lung cancer [8], (c) available clinicopathological and pretreatment laboratory data, (d) Eastern Cooperative Oncology Group performance status (ECOG-PS) of 0 − 1, and (e) no prior history of lung cancer or no intervention for lung cancer including surgery, chemotherapy, and radiotherapy. The exclusion criterion was the presence of hematologic disorder and advanced cancer.

### Clinical data collection

Using an electronic medical record system, we extracted relevant demographic data, ECOG-PS baseline values, pathological diagnosis, stage, and PD-L1 expression, as well as therapeutic data.

### Nutritional assessment using PNI

PNI was calculated based on a peripheral blood sample and using the formula:

10 × serum albumin (g/dl) + 0.005 × lymphocyte count (cells/mm^3^) (3). The peripheral blood obtained within 3 days immediately preceding durvalumab treatment.

### Statistical analysis

EZR (Saitama Medical Center, Jichi Medical University) was used to conduct all statistical tests in this paper [9], which is a user interface for R (ver. 4.0.3). Variables were presented using median (range). Fisher’s exact test was selected for categorical data, while Mann–Whitney *U*-test or Kruskal–Wallis one-way analysis was for continuous one. The receiver operating characteristic (ROC) curve determined the cutoff PNI value for predicting OS. PFS was considered the time from the first durvalumab dose to the disease progression or death date and censored on the day of the last disease assessment. Conversely, OS was considered the time from treatment to death date from any cause, with censoring on the last known alive date. OS and PFS parameters were calculated using Kaplan–Meier survival analysis, and the survival differences were calculated using a log-rank test. For identification of prognostic factors for survival, after testing the proportional hazard assumption, univariate and multivariate Cox proportional hazards regression analyses were performed to estimate hazard ratios (HRs) and 95% confidence intervals (CI).

Furthermore, univariate logistic regression analysis was selected to determine which factors affect PFS and OS. The chosen factors for analysis were as follows: age, sex, ECOG-PS, smoking history, pathology, stage, chemotherapy, PD-L1 expression, and PNI. Finally, based on the results of the univariate analysis, significant variables (*p*-value < 0.05) were included in the multivariate logistic regression analysis. All statistical tests were two-sided tests.

## Results

### Patient characteristics

Table 1 displays clinical characteristics. The median age of the 56 patients was 70 years (41–84), with 47 being men, 23 (41.1%) having adenocarcinoma, and 28 (50.0%) had squamous cell carcinoma. In terms of ECOG-PS, 44 (78.6%) and 12(21.4%) patients received a score of 0 and 1, respectively. There were 22 (39.3%), 30 (53.6%), and 4 (7.1%) patients with stage IIIA, IIIB, and IIIC, respectively.

**Table 1.** The clinical characteristics of patients with Stage III nonsmall cell lung cancer.

	All *n* = 56	Low-PNI *n* = 28	High-PNI *n* = 28	*p*-Value
Gender				
Male/female	47/9	25/3	22/6	0.469
Age				
Median (range), years	70 (41–84)	72 (64–79)	67 (41–84)	0.009*
Smoking status				
Never/ever or current	5/51	0/28	5/23	0.051
ECOG-PS				
0/1	44/12	18/10	26/2	0.020*
Stage				
IIIA/IIIB/IIIC	22/30/4	8/18/2	14/12/2	0.259
Pathology				
Adenocarcinoma/Squamous/others	23/28/5	8/18/2	15/10/3	0.113
Chemotherapy				
Carboplatin/Cisplatin	49/7	25/3	24/4	0.999
PD-L1 expression				
1%</1%–49%/≤50%	12/19/17	8/11/6	4/8/11	0.221

ECOG-PS: Eastern Cooperative Oncology Group performance status; PD-L1: programmed cell death 1 - ligand 1; PNI: prognostic nutritional index.

*p-Values < 0.05.

### Oncological outcomes in the entire cohort

The median follow-up period was 17.6 months (range, 3.0–45.4). Overall, 34 patients showed evidence of progression, and 19 died during the follow-up period. The median PFS and OS of all patients were 12.6 months (95% CI, 6.7–21.3) and 32.5 months (95% CI, 22.9 to not reached), respectively.

### Independent factors associated with oncological outcomes

According to univariate analysis, age, gender, ECOG-PS, smoking, pathology, stage, chemotherapy, PD-L1 expression, and PNI significantly affected OS and PFS (Table 2). ECOG-PS, pathology, PD-L1 expression, and PNI were identified as significant OS predictors. Table 3 shows the results of the multivariate Cox hazards regression analyses of oncological outcomes after adjusting for ECOG-PS, pathology, PD-L1 expression, and PNI. PNI value can significantly predict the OS of the patients (HR, 0.187; 95% CI, 0.046–0.760; *p* = 0.019).

**Table 2.** Univariate analysis of several parameters as predictors of PFS and OS in patients with stage III NSCLC.

		PFS			OS		
		HR	95% CI	*p*-Value	HR	95% CI	*p*-Value
Age	70</≤70	1.319	0.667–2.604	0.425	1.872	0.749–4.674	0.179
Gender	Male/Female	1.128	0.435–2.918	0.805	4.745	0.625–36.010	0.132
ECOG-PS	0/1	2.048	0.950–4.415	0.067	4.331	1.558–12.040	0.004*
Smoking	never/ever	0.992	0.301–3.268	0.990	3.800	0.495–29.150	0.199
Pathology	Non squamous/Squamous	1.963	0.926–4.160	0.078	3.402	1.12–10.331	0.031*
Stage	IIIA/IIIB/IIIC	0.953	0.552–1.645	0.862	1.230	0.597–2.531	0.574
Chemotherapy	Carboplatin/Cisplatin	0.761	0.267–2.166	0.608	0.681	0.156–2.971	0.609
PD-L1 expression	1%</1%–49%/≤50%	0.682	0.436–1.067	0.093	0.452	0.238–0.860	0.015*
PNI	37.9</≤37.9	0.459	0.229–0.919	0.027*	0.187	0.066–0.529	0.001*

ECOG-PS: Eastern Cooperative Oncology Group performance status; PD-L1: programmed cell death 1 – ligand 1; PNI: prognostic nutritional index; HR: hazard ratio; PFS: progressive free survival; OS: overall survival.

*p-Values < 0.05.

**Table 3.** Multivariate analysis for parameters as predictors of PFS and OS in patients with stage III NSCLC.

		PFS			OS		
		HR	95% CI	*p*-Value	HR	95% CI	*p*-Value
ECOG-PS	0/1	2.483	0.882–6.989	0.084	2.672	0.756–9.442	0.127
Pathology	Non squamous/Squamous	1.657	0.679–4.039	0.265	3.290	0.810–13.360	0.095
PD-L1 expression	1%</1%–49%/≤50%	0.940	0.241–1.298	0.176	1.105	0.504–2.423	0.803
PNI	37.9</≤37.9	0.559	0.241–1.298	0.176	0.187	0.046–0.760	0.019*

ECOG-PS: Eastern Cooperative Oncology Group performance status; PD-L1: programmed cell death 1 - ligand 1; PNI: prognostic nutritional index; HR: hazard ratio; PFS: progressive free survival; OS: overall survival.

*p-Values < 0.05.

### Identification of PNI cutoff values

**Figure 1.**
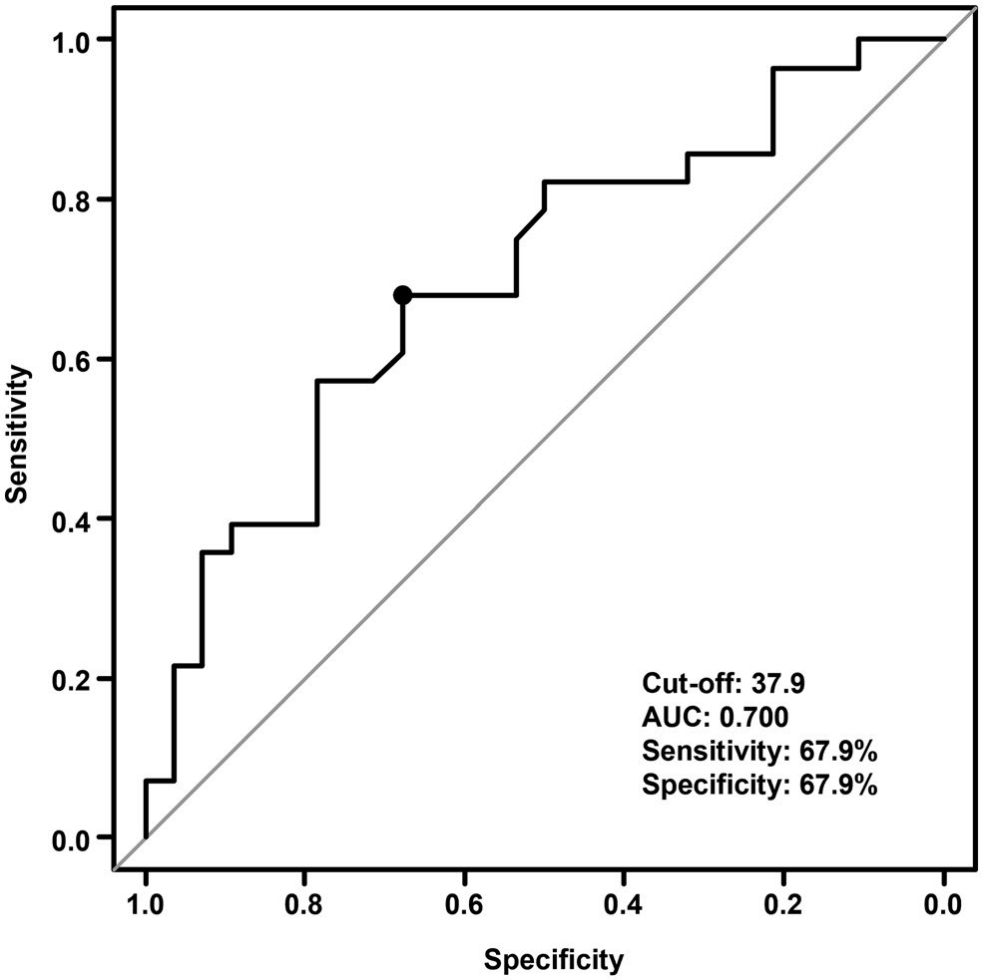
PNI ROC curves analysis for OS in stage III NSCLC patients. ROC: receiver operating characteristic; PNI: prognostic nutritional index; OS: overall survival; NSCLC: non-small cell lung cancer.

PNI was measured on median 9.5 days (range, 3.0–55.0) after chemoradiation. According to the ROC curve and Youden index, the pretreatment PNI cutoff value to predict OS was 37.9, and the area under the curve was 0.70 (95% CI: 0.56–0.84) (Figure 1). However, the specificity and sensitivity of PNI cutoff values were 67.9% and 67.9%, respectively. According to the PNI cutoff value, patients were divided into low-PNI (PNI ≤ 37.9) and high-PNI (PNI > 37.9) groups. Table 1 is a comparison of patient characteristics according to PNI. The low PNI group was significantly correlated with elderly and poor ECOG-PS.

### Oncological outcomes in low-PNI and high-PNI groups

**Figure 2.**
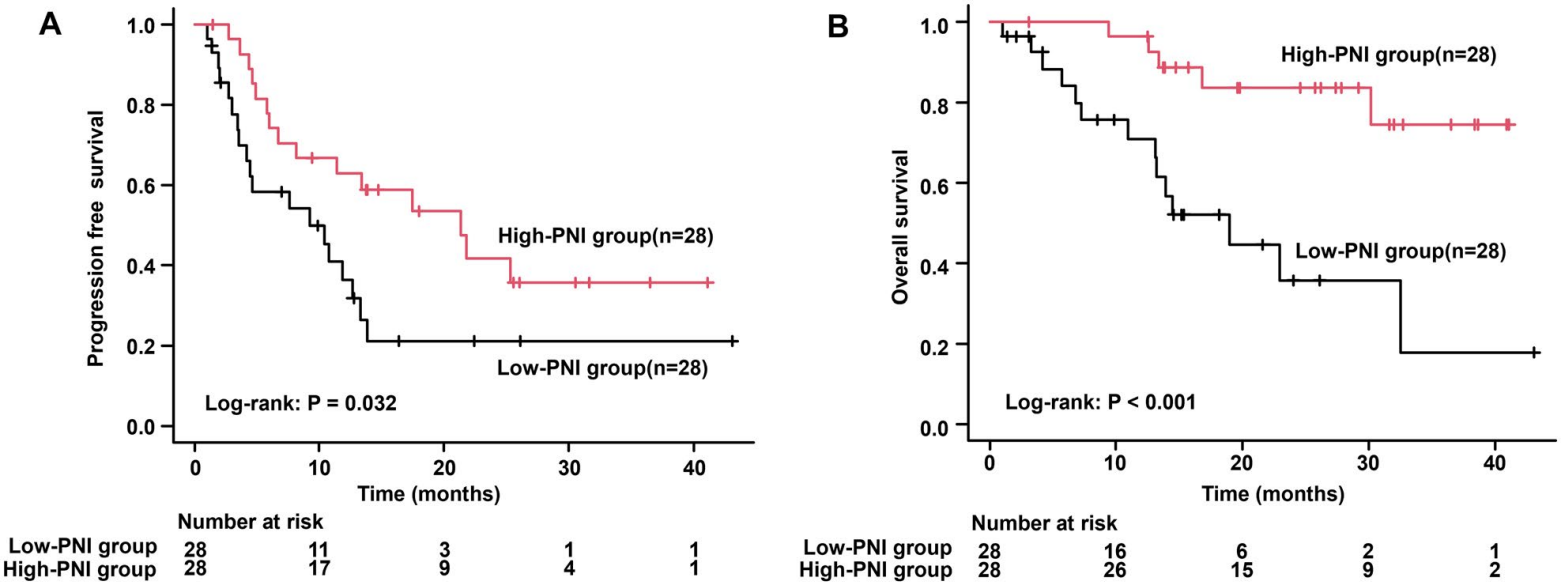
Based on PNI, a Kaplan–Meier curve analysis of PFS and OS for 56 NSCLC patients treated with durvalumab after chemoradiation therapy was performed. (A) PFS based on PNI. (B) OS based on PNI. PFS: progressive free survival; OS: overall survival; PNI: prognostic nutritional index; NSCLC: non-small cell lung cancer.

We investigated how PNI could influence the OS and PFS in the included patients. Low-PNI group had a significantly shorter PFS compared to the high-PNI group (median, 9.1 vs. 21.3 months, *p* = 0.032; Figure 2(A)). OS was also shorter in the low-PNI group (median, 19.0 months vs. not reached, *p* < 0.001; Figure 2(B)). As a result, patients with low PNI had significantly worse PFS and OS than those with high PNI.

## Discussion

This study looked at the predictive power of PNI as a measure of immunity and nutrition in stage III NSCLC patients. According to our results, PNI could be considered an independent predictor for the survival of stage III NSCLC patients treated with durvalumab after chemoradiation therapy. Consequently, pretreatment immune and nutritional assessment using PNI are important to evaluate the prognosis of those patients.

The immunological alterations due to chemoradiation therapy should be considered when predicting durvalumab outcomes. Blood components, including lymphocytes, can be directly affected by chemotherapy-induced myelosuppression and radiation to the spinal cord. Albumin synthesis may decrease due to anorexia caused by chemotherapy and inflammation by radiotherapy, which is consequently associated with short survival [10]. PNI is calculated as a prognostic factor for cancer using lymphocyte count and serum albumin [3]. We had previously shown that low PNI before first-line therapy was an important indicator of poor OS in patients with advanced NSCLC [11]. In the present study, lower PNI before durvalumab was also associated with shorter survival. Inadequate immunocompetence and nutritional status after chemoradiation therapy may result in poor antitumor efficacy of ICIs.

The level of PD-L1 expression in tumor specimens before treatment for advanced lung cancer is considered a predictive marker of the antitumor efficacy of ICI [12–13]. It remains uncertain whether PD-L1 expression in tumor specimens before chemoradiation therapy is a predictive marker for antitumor efficacy of ICI after chemoradiation. The antitumor efficacy is highly dependent on the patient’s immune competence. A previous study showed that PNI level before treatment was a prognostic factor for NSCLC patients on ICI therapy [14]. After chemoradiation therapy, our results show that the low PNI before durvalumab treatment is an independent predictor of OS in patients with stage III NSCLC. PNI provides a measure of both immunological and nutritional status in patients with malignancies and warrants further studies as a predictive marker of ICI.

The first limitation of this study is the retrospective design, and the modes or small sample size. It is thought that this is because it is a single institution, and we carefully selected patients with stage III NSCLC who had undergone chemoradiation therapy without severe side effects or progression and who could be administered durvalumab. Therefore, a study with a larger sample size, standardized treatment, and a longer follow-up period should be conducted to validate the significance of PNI. Second, we did not perform PD-L1 testing in all cases. PD-L1 expression has been shown to be effective for ICI in advanced NSCLC [12,13], but the effectiveness of ICI in stage III NSCLC is uncertain and is not an essential test. Third, we only investigated the PNI for predicting the efficacy of durvalumab and did not examine other immunological and nutritional indexes. PNI, calculated from lymphocyte counts and serum albumin, appears convenient and reasonable for predicting ICI response.

To summarize, this is the first study to look at the prognostic impact of PNI on survival in patients with stage III NSCLC receiving durvalumab after chemoradiation therapy.

## Data Availability

The datasets used and analyzed during the current study are available from the corresponding author on a reasonable request.
